# Smooth transformation models for survival analysis: A tutorial using R

**DOI:** 10.1177/09622802251414595

**Published:** 2026-04-27

**Authors:** Sandra Siegfried, Bálint Tamási, Torsten Hothorn

**Affiliations:** 1 Institut für Epidemiologie, Biostatistik und Prävention, Universität Zürich, Switzerland

**Keywords:** Non-proportional hazards, dependent censoring, clustered observations, personalized medicine, survival trees, R

## Abstract

Over the last five decades, we have seen strong methodological advances in survival analysis, using parametric methods and, more prominently, methods based on non-/semi-parametric estimation. As the methodological landscape continues to evolve, the task of navigating through the multitude of methods and identifying available software resources is becoming increasingly challenging—especially in more complex scenarios, such as when dealing with interval-censored or clustered survival data, non-proportional hazards, or dependent censoring. This tutorial explores the potential of using the framework of smooth transformation models for survival analysis in the R system for statistical computing. This framework provides a unified maximum-likelihood approach that covers a wide range of survival models, including well-established ones such as the Weibull model and a fully parametric version of the famous Cox proportional hazards model, and various extensions for more complex scenarios. We explore models for non-proportional/crossing hazards, dependent censoring, clustered observations and extensions towards personalized medicine within this framework. Using survival data from a two-arm randomized controlled trial on rectal cancer therapy, we demonstrate how survival analysis tasks can be seamlessly navigated in R within this framework using the implementation provided by the **tram** package, and few related packages.

## 1. Introduction

In “parametric” survival analysis, researchers commonly rely on the Weibull model or alternative accelerated failure time models. To achieve greater flexibility and overcome the strict distributional assumptions underlying these models, researchers often need to turn to non-/semi-parametric methods to analyze their survival data. When it comes to non-/semi-parametric approaches, however, overcoming issues tied to interval-censored or truncated observations can prove challenging due to their limited availability in standard software implementations.

Furthermore, when aiming to fit models that handle crossing or non-proportional hazards, clustered observations, or dependent censoring, researchers often find themselves navigating a complex landscape of diverse software implementations. Even the same models can be difficult to compare across different implementations, because different parameterizations, estimation strategies or optimization procedures are used.

This becomes even more challenging when comparing different models computed from different packages—emphasizing the benefit of a unified framework that facilitates seamless transitions between different models and is based on a common core infrastructure for model parameterization, estimation strategy, and optimization procedure.

To tackle these challenges, researchers may explore the potential of the **tram** add-on package^1^ in R,^2^ which offers a flexible framework for survival analysis. The **tram** package implements a user-friendly interface to smooth transformation models,^3^ which encompass a range of classical survival models including accelerated failure models and the Cox proportional hazards model, as well as many useful extensions and novel model formulations. The package can be easily installed from the Comprehensive R Archive Network (CRAN):


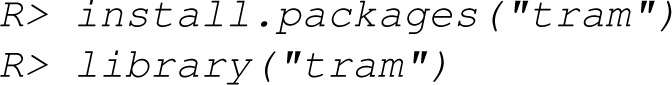


All models are implemented in a fully parametric fashion, allowing for straightforward maximum likelihood inference. The estimation of these models is facilitated by the core infrastructure package **mlt**^4,5^ which provides a unified maximum likelihood framework for general transformation models.^3^ We further leverage two add-on packages from this family of packages for transformation modeling: the **tramME** package^6,7^ implementing mixed-effects and non-linear additive effects for smooth transformation models, and the **trtf** package^8^ for estimating tree-based survival models.

In this tutorial, we will explore a variety of models commonly utilized in survival analysis. The focus of this tutorial lies on the practical implementation and interpretation of these models within the framework of smooth transformation models, rather than on theoretical aspects. Our objective is to provide users with a practical understanding of how to apply these models using R. Through an application to data from a randomized trial on rectal cancer therapy, we showcase how users can seamlessly conduct their survival analysis tasks without the need to navigate through a multitude of packages in R.

In Section 3, we discuss models for independent observations. We start with the well-known Weibull model, and then, to introduce more flexibility and overcome the log-linear log-cumulative hazard assumption inherent to the Weibull model, we explore a fully parametric version of the Cox model. We further discuss the estimation of stratified log-cumulative hazard functions to account for baseline risk variations across patient strata. Moving beyond the assumption of proportional hazards, we showcase models that challenge this assumption. We discuss a location-scale version of the Cox model, accommodating scenarios with non-proportional/crossing hazards, and models estimating time-varying treatment effects.

Addressing scenarios where the assumption of independent censoring might not be justified, we discuss a copula proportional hazards model, that accommodates dependent censoring (Section 4). For clustered observations we employ mixed-effects Cox models and alternative models featuring marginally interpretable hazard ratios in Section 5. Our tutorial also explores the domain of personalized medicine, presenting models that incorporate covariate-dependent treatment effects and survival trees (Section 6). In Section 7, we explore further extensions, including topics like frailty models, model estimation using the non-parametric likelihood, covariate adjustment and the potential of using these models for sample size estimation of new trials.

This tutorial is composed of the main text, which introduces the models and very briefly shows how to estimate them using smooth transformation models in R. In addition, we present head-to-head comparisons of user-interfaces and numerical results obtained from alternative packages available in the R universe in Supplementary Material A. Both parts come with much more detailed R code for exploring fitted models (for example, plotting model terms, computing confidence intervals, or performing tests), which can be explored in the corresponding demo:


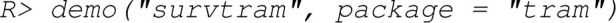


In our Supplementary Material A, we conduct a thorough comparison of a subset of the models discussed here which can be estimated using alternative implementations (in total 13 established CRAN packages) and corresponding results obtained with smooth transformation models from **tram** and **tramME**. This quality assurance task not only helped to validate the implementation in **tram** and **tramME** but also led to the identification of problematic special cases and, in some instances, practically relevant discrepancies between different package implementations of the very same model. Moreover, Supplementary Material A presents the different user-interfaces of the different packages side-by-side, such that it becomes simpler to estimate and compare relatively complex models across independent implementations. For the analysis of future survival trials, an assessment of the agreement of such estimates, standard errors, and possibly other model aspects can help to increase trust in reported numerical results and conclusions derived therefrom.

## 2. Application

In our tutorial, we will work with data from the CAO/ARO/AIO-04 two-arm randomized controlled trial,^9^ a phase 3 study that aimed to compare an experimental regimen with the previously established treatment regimen (control) for locally advanced rectal cancer. In this experimental regimen, Oxaliplatin was added to the control treatment of preoperative Fluorouracil-based chemoradiotherapy and postoperative chemotherapy of locally advanced rectal cancer.

The trial was conducted across 88 centers and included a cohort of 1236 patients. The patients were randomly allocated to the two treatment arms W∈{0,1} , receiving the experimental treatment of Oxaliplatin added to Fluorouracil-based preoperative chemoradiotherapy and postoperative chemotherapy (5-FU + Oxaliplatin, W=1 ) or the control treatment using Fluorouracil only (5-FU, W=0 ). Treatment allocation was performed using block-randomization stratified by study center j=1,…,88 and the stratum s , which is defined by four categories consisting of a combination of clinical N category, *i.e.,* lymph node involvement (cN0 vs cN+), and clinical T category *i.e.,* tumor grading (cT1-3 vs cT4). The distribution of patients in the two treatment arms across strata is shown in Table 1.

**Table 1.** Number of patients in each treatment arm stratified by the combination of clinical N and T category.

	5-FU	5-FU + Oxaliplatin
cT1-3 : cN0	163	156
cT4 : cN0	8	7
cT1-3 : cN+	411	417
cT4 : cN+	41	33
Total	623	613

**Figure 1.**
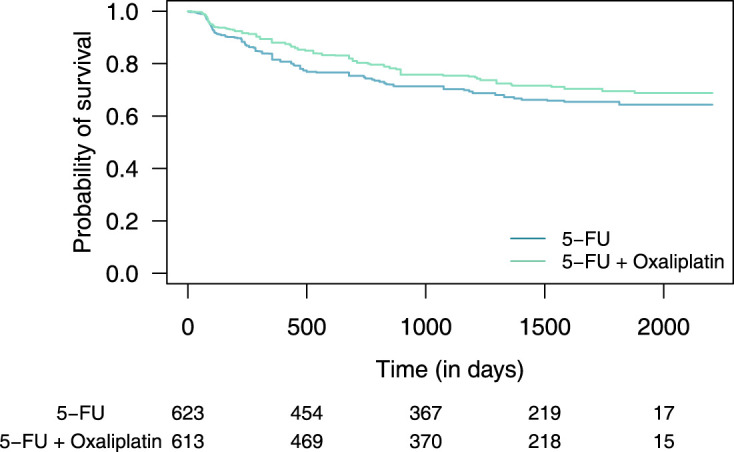
Disease-free survival. The survivor functions of the two treatment arms estimated by the non-parametric Turnbull method are shown together with the number at risk table.

The primary endpoint is disease-free survival, defined as the time $T\in R^{+}$ between randomization and non-radical surgery of the primary tumor (R2 resection), loco-regional recurrence after R0/1 resection, metastatic disease or progression, or death from any cause—whichever occurred first. The observed times encompass a mix of exact observations t for time to death or incomplete removal of the primary tumor, interval-censored observations $t\in (\underline{t},\bar{t}]$ for the time span from the previous follow-up $\underline{t}$ to the time-point of detecting local or distant metastases $\bar{t}$ , and right-censored observations t∈(t,∞) corresponding to the end of the follow-up period or instances where patients were lost to follow-up. The survivor curves of the primary endpoint (disease-free survival) estimated by the non-parametric Turnbull estimator^10^ are shown for the two treatment arms in Figure 1.

**Figure 2.**
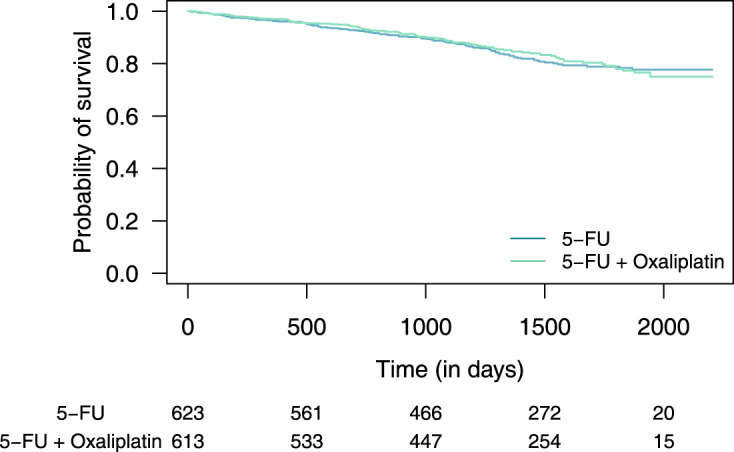
Overall survival. The survivor functions of the two treatment arms estimated by the non-parametric Kaplan–Meier method are shown together with the number at risk table.

A secondary endpoint considered in the study is overall survival, defined as the time $T\in R^{+}$ from randomization to death from any cause. Notably, all observations t for this endpoint are exact or right-censored. The corresponding survivor curves, estimated non-parametrically by the Kaplan–Meier method,^11^ are shown in Figure 2.

The primary data analysis for this trial was performed by Rödel et al.^9^ In their analysis, the treatment effect comparing the effect of the experimental treatment to the effect of the control treatment on disease-free and overall survival was assessed by adjusted log-rank tests and mixed-effects Cox models (both adjusting for the stratified randomization process), treating both survival endpoints as exact. Following, we demonstrate the process of fitting a fully parametric mixed-effects Cox model that accounts for interval-censored event times of the primary endpoint in Section 5, also in terms of a model featuring marginal interpretation of the estimated hazard ratio.

A subsequent post hoc analysis was carried out by Hofheinz et al.^12^ In this analysis, the possibility of an age-varying treatment effect on both the primary endpoint of disease-free survival and the secondary endpoint of overall survival was investigated. In Section 6, we demonstrate how such an analysis can be performed within the discussed framework, taking into account interval-censoring. We illustrate two approaches for estimating age-varying effects, using smooth age-varying hazard ratios and age-structured survival trees.

While the analyses conducted by Rödel et al.^9^ and Hofheinz et al.^12^ made serious efforts to address the research questions effectively, they were limited by the lack of software resources capable of adequately handling interval-censored and correlated observations for the analysis of the primary endpoint. Notably, the first R add-on package capable of estimating Cox models in the presence of interval-censoring was published in 2014 (**coxinterval** package^13^). At the time of the statistical analysis of the primary endpoint, it was impossible to fit mixed-effects models with flexible baseline hazards to interval-censored outcomes. This obstacle was one of the main motivation to develop a comprehensive software package implementing a general class of transformation models with applications in the domain of survival analysis. The corresponding framework implementing smooth transformation models^3^ in R helps to address such and other practically relevant limitations. In this tutorial, we present analyses that the CAO/ARO/AIO-04 study investigators would have liked to have been able to perform a decade ago.

## 3. Independent observations

### 3.1. Survival models

#### 3.1.1. Weibull proportional hazards model

Probably one of the most renowned parametric model in survival analysis is the Weibull model,^14^ where the response T conditional on treatment assignment W=w is assumed to follow a Weibull distribution. We consider the Weibull proportional hazards model with survivor functions conditional on the treatment assignment parameterized as,

$$\begin{array}{rl} S_{0}(t) & =P(T>t|W=0)=\exp\;\left\{-\exp\;\left[\vartheta_{1}+\vartheta_{2}\log\;(t)\right]\right\},\;\vartheta_{2}>0,\\ S_{1}(t) & =P(T>t|W=1)=\exp\;\left\{-\exp\;\left[\vartheta_{1}+\vartheta_{2}\log\;(t)-\beta \right]\right\}, \end{array}$$

with the general formula,

(1) $$S_{w}(t)=P(T>t|W=w)=\exp\;\left\{-\exp\;\left[\vartheta_{1}+\vartheta_{2}\log\;(t)-\beta w\right]\right\}.$$

The log-cumulative baseline hazard $\log\;(-\log\;(S_{0}(t)))=\log\;(\Lambda_{0}(t))$ here is given by $h(t)=\vartheta_{1}+\vartheta_{2}\log\;(t)$ , assuming a linear shift β on the scale of log-time log(t) . The model not only assumes proportional hazards, with hazard ratio $\frac{\Lambda_{1}(t)}{\Lambda_{0}(t)}=\frac{\lambda_{1}(t)}{\lambda_{0}(t)}=\exp\;(-\beta )$ , but is also an accelerated failure time model

$$\begin{array}{rl} \log\;(T) & =\frac{-\vartheta_{1}+\beta w+Z}{\vartheta_{2}},\;Z∼\mathrm{MEV}\\ & =-\frac{\vartheta_{1}}{\vartheta_{2}}+\frac{\beta }{\vartheta_{2}}w+\frac{1}{\vartheta_{2}}Z=\alpha +\tilde{\beta }w+\sigma Z, \end{array}$$

with the errors Z following a minimum extreme value distribution (MEV). Consequently, T∣W=w follows a Weibull distribution (Kalbfleisch and Prentice^15^, Chapter 2) with intercept $\alpha =-\vartheta_{1}\vartheta_{2}^{-1}$ , scale parameter $\sigma =\vartheta_{2}^{-1}$ and log-acceleration factor $\tilde{\beta }=\beta \vartheta_{2}^{-1}$ . The model implies that, for the experimental arm time T is accelerated by $\exp\;(\tilde{\beta })$ , that is $T_{1}=\exp\;(\tilde{\beta })T_{0}$ , thus the probability of disease-free survival for the experimental arm is given by $S_{1}(t)=S_{0}(\exp\;(-\tilde{\beta })t)$ .

Alternatively, different distributions for Z , such as the normal or logistic distribution, can be specified, leading to the formulation of log-normal or log-logistic models.

Parameter estimation of the Weibull model is straightforward using maximum likelihood, because the distribution function can be directly evaluated and thus allows to effectively handle interval-censored or truncated data, as will be discussed in Section 3.2.

#### 3.1.2. Flexible proportional hazards model

The assumption of a log-linear log-cumulative baseline hazard function h , implied by the Weibull model, can be relaxed by replacing $\log\;(\Lambda_{0}(t))=h(t)=\vartheta_{1}+\vartheta_{2}\log\;(t)$ with a more flexible function $h(t)=a{\left(t\right)}^{⊤}\vartheta$ defined in terms of smooth spline basis functions a and corresponding parameters ϑ . This yields the following model

(2) $$S_{w}(t)=P(T>t|W=w)=\exp\;\left\{-\exp\;\left[h(t)+\beta w\right]\right\},$$

where the hazard ratio is given by $\frac{\Lambda_{1}(t)}{\Lambda_{0}(t)}=\frac{\lambda_{1}(t)}{\lambda_{0}(t)}=\exp\;(\beta )$ . McLain and Ghosh^16^ and Hothorn et al.^3^ proposed parameterizing $h(t)=a{\left(t\right)}^{⊤}\vartheta$ as polynomials in Bernstein form $h(t)=a_{\text{Bs},P-1}{\left(t\right)}^{⊤}\vartheta$ of order P−1 . The corresponding model (2) is a fully parametric version of the otherwise semi-parametric Cox proportional hazards model.^17^ The latter treats h as an infinite dimensional nuisance parameter which is profiled out from the likelihood. This leads to the partial likelihood, through which the log-hazard ratio β can be estimated.^18^ In contrast, all parameters of model (2) are estimated simultaneously by maximum likelihood. The parameterization of h in terms of basis functions and corresponding parameters allows to specify of a flexible, yet fully parametric, monotonically increasing log-cumulative hazard function. This is achieved under appropriate constraints $\vartheta_{p}\leq \vartheta_{p+1}$ for p∈1,…,P−1 .^3^ Adopting this specific parameterization for the log-cumulative baseline hazard function $\log\;(\Lambda_{0}(t))=h(t)$ facilitates the computation of the corresponding density $f_{0}(t)$ and distribution function $F_{0}(t)$ , thus allowing for straightforward parameter estimation using maximum likelihood. This holds true even when dealing with interval-censored or truncated observations.

Within the **tram** add-on package, the order, P−1 , of polynomials in Bernstein form is not determined in a data-driven way. The default P−1=6 is typically a good compromise between flexibility of h(t) and computing time needed to optimize the log-likelihood. Fixed P also facilitates standard maximum likelihood inference. Because of the monotonicity constraint on h , the transformation function h it not prone to overfit and thus, in principle, P can be chosen much larger. The effect of larger P on estimates of other model parameters and their standard errors is very small (see, e.g. the log-hazard ratios in Figure 5 provided by Hothorn^4^ and the empirical comparison to non-parametric models^19^). However, if one is interested in expressions involving the derivative $h^{\prime }(t)$ , which itself is in Bernstein form, the order P−1 must be chosen in a data-driven way, for example for density estimation. Sieve maximum likelihood procedures have been suggested in this context, for example in Cox models with log-cumulative baseline hazard functions in Bernstein form.^16^

#### 3.1.3. Stratified proportional hazards model

Accounting for variations in baseline risks among different patient strata identified by variable s , one can employ stratified models that incorporate stratum-specific log-cumulative baseline hazard functions h(t∣s) . These models can be defined by

$$S_{w}(t|s)=P(T>t|S=s,W=w)=\exp\;\left\{-\exp\;\left[h(t|s)+\beta w\right]\right\},$$

with $h(t|s)=a{\left(t\right)}^{⊤}\vartheta (s)$ and global hazard ratio $\frac{\Lambda_{1}(t|s)}{\Lambda_{0}(t|s)}=\frac{\lambda_{1}(t|s)}{\lambda_{0}(t|s)}=\exp\;(\beta )$ , assuming that the treatment effect is the same across all patient strata s .

One could further relax the restriction of a global treatment effect, allowing for an interaction of the treatment assignment W=w and the stratum s by formulating the log-cumulative hazard as $h(t|s)+w\beta +\gamma^{⊤}(w\times s)$ .

#### 3.1.4. Non-proportional hazards model

Extending beyond the proportional hazards assumption, an additional treatment-dependent model term can be estimated. Burke and MacKenzie^20^ introduced the multiparameter extension to the Weibull model (1) in the context of survival analysis, specifically outlining its use for interval-censored observations.^21^

A similar extension can be made to the more flexible, parametric, Cox model (2), by additionally estimating a scale term γ for the experimental arm,^22^

$$S_{w}(t)=P(T>t|W=w)=\exp\;\left\{-\exp\;\left[\sqrt{\exp\;(\gamma w)}\;h(t)+\beta w\right]\right\}.$$

In this case the ratio of the cumulative hazards, $\frac{\Lambda_{1}(t)}{\Lambda_{0}(t)}$ , is a non-proportional function of t . The corresponding bivariate score test (Section 3.1.2 of Siegfried et al.^22^) further allows to test the null hypothesis of equal survival, *i.e.,* β=γ=0 , without relying on the assumption of proportional hazards.

#### 3.1.5. Time-varying hazards model

Accounting for changing effects of the treatment over time, we can further extend beyond the proportional hazards assumption and estimate a model incorporating a time-varying treatment effect,

$$S_{w}(t)=P(T>t|W=w)=\exp\;\left\{-\exp\;\left[h(t)+\beta (t)w\right]\right\}.$$

Here, the model introduces a time-varying shift β(t) in the log-cumulative hazard function $\log\;(\Lambda_{1}(t))=\log\;(\Lambda_{0}(t))+\beta (t)$ , thereby relaxing the assumption of a constant log-hazard ratio β . The shift β(t) is also parameterized in terms of a polynomial in Bernstein form, thus allowing to estimate a time-varying ratio of the cumulative hazards $\frac{\Lambda_{1}(t)}{\Lambda_{0}(t)}=\exp\;(\beta (t))$ .

### 3.2. Likelihood

From the above models, the log-likelihoods for exact or independently right-, left- or interval-censored and truncated observations are easily deducible. We here consider the most general case where the log-cumulative hazard function is given by $h(t|w,s)=\sqrt{\exp\;(\gamma w)}\;h(t|s)+\beta w$ .

For exact continuous observations (t,w,s) , the corresponding log-likelihood contributions are given by

$$\ell (\vartheta (s),\beta ,\gamma |T=t)=\log\;\left\{f\left[h(t|w,s)\right]\right\}+\log\;\left\{h^{\prime }(t|w,s)\right\},$$

with the standard minimum extreme value density f(z)=exp(z−exp(z)) and the derivative of the log-cumulative hazard function with respect to t , $h^{\prime }(t|w,s)=\sqrt{\exp\;(\gamma w)}\;a^{\prime }{\left(t\right)}^{⊤}\vartheta (s)$ .

Because the transformation function h , defining the log-cumulative baseline hazard function, is parameterized in terms of polynomials in Bernstein form, where the basis functions $a_{\text{Bs},P-1}(t)\in R^{P}$ are specific beta densities,^23^ it is straightforward to obtain the derivatives of the basis functions with respect to t , leading to $h^{\prime }(t|s)=a_{\text{Bs},P-1}^{\prime }{\left(t\right)}^{⊤}\vartheta (s)$ .

Under independent left-, right- or interval-censored event times $\left(\underline{t},\bar{t}\right]$ the exact log-likelihood contribution is

$$\ell (\vartheta (s),\beta ,\gamma |T\in (\underline{t},\bar{t}])=\log\;\left\{P(T\in (\underline{t},\bar{t}]|w,s)\right\}=\log\;\left\{S_{w}(\underline{t}|w,s)-S_{w}(\bar{t}|w,s)\right\}.$$

For observations that are right-censored at time t the datum is given by $\left(\underline{t},\bar{t}]=(t,\infty \right)$ and for left-censored observations it is $\left(\underline{t},\bar{t}]=(0,t\right]$ .

In cases where event times are subject to random left-, right-, or interval-truncation $(t_{\text{l}},t_{\text{r}}]\subset R^{+}$ , the above log-likelihood contributions change to

$$\ell (\vartheta (s),\beta ,\gamma |T\in (\underline{t},\bar{t}])-\ell (\vartheta (s),\beta ,\gamma |T\in (t_{\text{l}},t_{\text{r}}])$$

with $t_{\text{l}}=0$ for right-truncated and $t_{\text{r}}=\infty$ for left-truncated observations. Such considerations are relevant in scenarios involving a late entry approach, for instance, resulting in left-truncated observations, where one is interested in modeling $P(T>t|T\in (t_{\text{l}},\infty ))$ , or for modeling time-varying covariates.

### 3.3. Application

Now, turning our attention to the CAO/ARO/AIO-04 two-arm randomized controlled trial, we aim to estimate the previously discussed models using the unified maximum likelihood framework provided by the R add-on package **tram**.^1^ We fit the models to the primary endpoint of disease-free survival T estimating the treatment effect corresponding to the assignment W=randarm .

The disease-free survival times are stored as iDFS, an object of class “Surv,” which includes a mix of exact, interval-, and right-censored observations. This “Surv” object can be specified with Surv(CAOsurv$iDFStime, CAOsurv$iDFStime2, type = "interval2").^24,25^ Exact observations are represented by two identical time points, for interval-censored observations, the two times define the period within which the event occurred and right-censored observations are represented by an interval from the last visit to infinity.

We start by fitting the Weibull model (Section 3.1.1) using the Survreg() function.


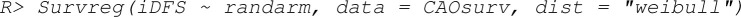


**Table:** 

Coefficient	Estimate	Std. Error	95%-Wald CI
$\vartheta_{1}$	−6.231	0.265	−6.752 to −5.711
$\vartheta_{2}$	0.733	0.036	−0.663 to −0.803
β	0.229	0.106	−0.020 to −0.438
Log-Likelihood	Likelihood Ratio Test	Score Test	Permutation Score Test
− 2281.17	p = 0.031	p = 0.031	p = 0.035

The model quantifies the treatment effect through a hazard ratio $\exp\;(-\hat{\beta })=0.795$ , comparing the hazards of the experimental arm to the hazards of the control arm. The results indicate reduced hazards for patients receiving the experimental treatment compared to the control treatment. This reduction in hazards corresponds to a prolonged disease-free survival time in the experimental arm. Since the model is a proportional hazards counterpart of the Weibull accelerated failure time model fitted by survreg() from the **survival** package,^24,25^ the estimate can also be translated into a log-acceleration factor $\hat{\tilde{\beta }}=\hat{\beta }{\hat{\vartheta }}_{2}^{-1}=0.312$ . This implies that the disease-free survival time T is prolonged by $\exp\;(\hat{\tilde{\beta }})=1.367$ in the experimental arm, compared to the control arm.

Next, we fit the flexible proportional hazards model (Section 3.1.2) using the Coxph() function from the **tram** package.


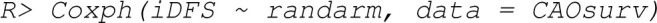


**Table:** 

Coefficient	Estimate	Std. Error	95%-Score CI
β	−0.231	0.107	−0.439 to −0.022
Log-Likelihood	Likelihood Ratio Test	Score Test	Permutation Score Test
− 2242.25	p = 0.030	p = 0.030	p = 0.030

**Figure 3.**
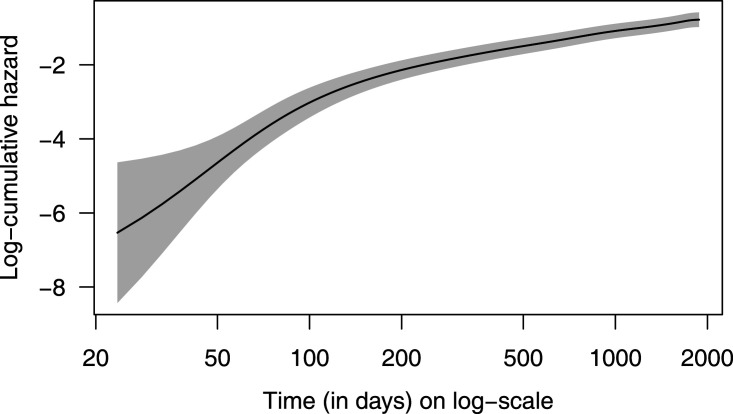
Flexible proportional hazards model. Log-cumulative baseline hazard function and corresponding 95%-confidence band estimated by the model.

The fitted model is a fully parametric version of the famous Cox model, otherwise estimated semi-parametrically using the partial likelihood (as implemented in the **survival** package in the coxph() function). Here the log-cumulative hazard function is specified in terms of polynomials in Bernstein form, by default of order P−1=6 , specifying the transformation function $h(t)=a_{\text{Bs},6}{\left(t\right)}^{⊤}\vartheta$ . The fully parametric approach enables the straightforward incorporation of interval-censored disease-free survival times. Figure 3 illustrates the estimated log-cumulative baseline hazard function $\log\;({\hat{\Lambda }}_{0}(t))=\hat{h}(t)=a_{\text{Bs},6}{\left(t\right)}^{⊤}\hat{\vartheta }$ along with the 95% -confidence band, revealing a nonlinear function of log-time. The band was obtained from simultaneous confidence intervals for a dense grid of time points utilizing the asymptotic normality of the maximum likelihood estimator $\hat{\vartheta }$ and the fact that h was parameterized as a contrast. The estimated hazard ratio is $\exp\;(\hat{\beta })=0.794$ , indicating reduced hazards in the experimental arm. The software implementation further allows to compute a corresponding 95%-permutation score confidence interval, which ranges from 0.645 to 0.978 .

To further accommodate for varying log-cumulative baseline hazard functions $\Lambda_{0}(t|s)$ across patient strata s (here identified by strat ), we can fit a stratified model (Section 3.1.3).


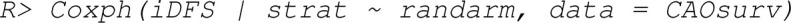


**Table:** 

Coefficient	Estimate	Std. Error	95%-Score CI
β	−0.228	0.107	−0.436 to −0.019
Log-Likelihood	Likelihood Ratio Test	Score Test	Permutation Score Test
− 2213.94	p = 0.031	p = 0.032	p = 0.033

**Figure 4.**
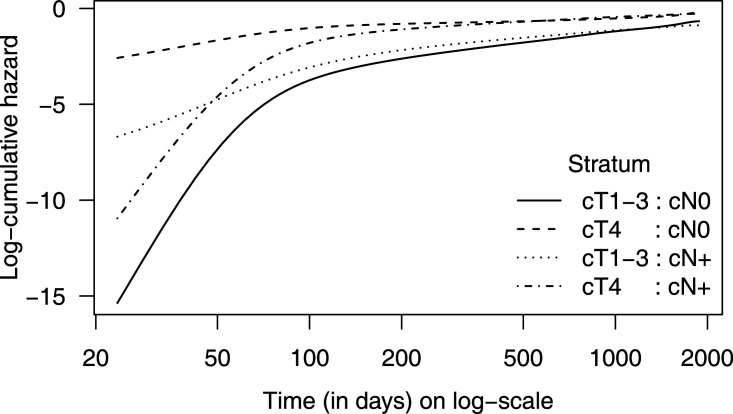
Stratified proportional hazards model. Log-cumulative baseline hazard functions $\Lambda_{0}(t|s)$ estimated by the model are shown separately for each stratum s .

The model estimates separate smooth log-cumulative baseline hazard functions for each stratum s , as illustrated in Figure 4, but provides an estimate of the global hazard ratio $\exp\;(\hat{\beta })=0.796$ , indicating a reduction of the hazard in the experimental arm by 0.796 relative to the hazard in the control arm across all stratum.

Moving away from the proportional hazards assumption, we can fit a non-proportional hazards model (a location-scale version of the Cox model, Section 3.1.4) using the same function.


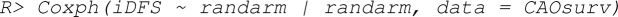


**Table:** 

Coefficient	Estimate	Std. Error	95%-Wald CI
β	−0.091	0.163	−0.411 to 0.229
γ	0.257	0.203	−0.140 to 0.654
Log-Likelihood	Likelihood Ratio Test	Bivariate Permutation Score Test	
− 2241.46	p = 0.043	p = 0.027	

**Figure 5.**
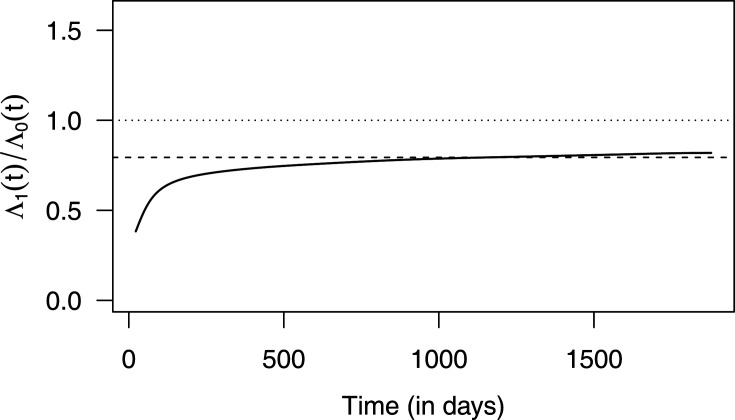
Non-proportional hazards model. $\frac{\Lambda_{1}(t)}{\Lambda_{0}(t)}$ (solid line) estimated by the model is shown alongside the constant hazard ratio estimated from the proportional hazards model (dashed line) over time t .

The ratio of the cumulative hazards $\frac{{\hat{\Lambda }}_{1}(t)}{{\hat{\Lambda }}_{0}(t)}$ , shown in Figure 5, no longer remains proportional but varies over time. The curve indicates a pronounced initial reduction in cumulative hazards for the experimental arm compared to the control arm, which gradually decreases as time progresses. This suggests that the treatment effect is stronger in the beginning. The corresponding bivariate score test, which tests the null hypothesis of equal survival without assuming proportional hazards, further indicates evidence for a difference in disease-free survival.

Finally, we fit the model featuring a time-varying treatment effect (Section 3.1.5).


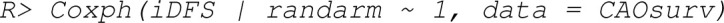


**Figure 6.**
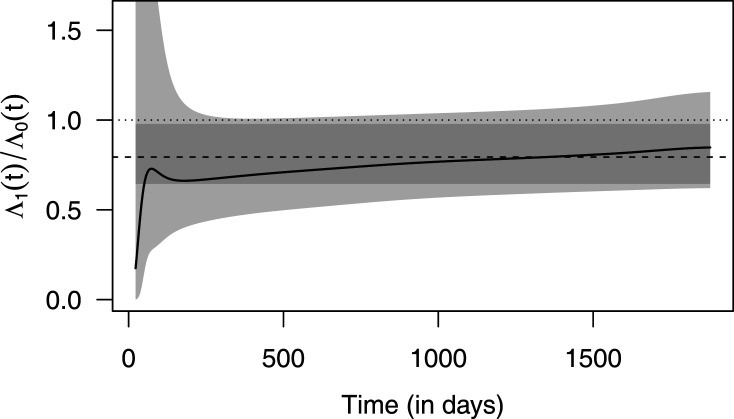
Time-varying hazards model. $\frac{\Lambda_{1}(t)}{\Lambda_{0}(t)}$ and corresponding 95%-confidence bands over time t (solid line) estimated by the model is shown alongside the constant hazard ratio estimated from the proportional hazards model (dashed line). The log-likelihood of the model is − 2240.21.

The treatment effect $\frac{\Lambda_{1}(t)}{\Lambda_{0}(t)}=\exp\;(\beta (t))$ is a function of time, as shown in Figure 6. The curve again demonstrates a reduction in hazards for the experimental arm compared to the control arm, which is more substantial in the beginning and gradually becomes less prominent as time progresses.

## 4. Dependent censoring

### 4.1. Copula proportional hazards model

Until now, the models, we have discussed, have been constructed under the assumption of independent censoring, which implies that the censoring times C are independent of the event times T given the treatment assignment W=w , that is T⊥⊥C∣W=w . We can however move beyond relying on this assumption and allow the model to capture potential dependence between the censoring times C and event times T .

We explore models discussed in recent work of Czado and Van Keilegom,^26^ which involve a joint model for T and C employing a bivariate Gaussian copula of the marginal survivor functions $S_{w}(t)$ and $G_{w}(c)$ of T and C respectively,

$$P(T\leq t,C\leq c|W=w)=\Phi_{0,R(\xi )}\left\{\Phi^{-1}\left[1-S_{w}(t)\right],\Phi^{-1}\left[1-G_{w}(c)\right]\right\}$$

with correlation matrix

$$\begin{array}{r} R(\xi )=\left[\begin{array}{cc} 1 & \frac{-\xi }{\sqrt{1+\xi^{2}}}\\ \frac{-\xi }{\sqrt{1+\xi^{2}}} & 1 \end{array}\right],\;\xi \in (-\infty ,\infty ) \end{array}$$

to account for the association between T and C . Deresa and Van Keilegom^27^ recently demonstrated that the above model maintains identifiability when the marginal survivor functions $S_{w}(t)$ and $G_{w}(c)$ are described by a flexible proportional hazards model (2) and a model that assumes a Weibull distribution (1), respectively. This allows to estimate the dependence parameter ξ and other model parameters from the observed data. A dependence parameter ξ of zero corresponds to uncorrelated event times T and censoring times C , thus indicating lack of evidence for dependent censoring.

### 4.2. Application

Returning to our application, where we previously assumed independent censoring of disease-free survival times, we now aim to address the potential scenario where loss of follow-up time $C\in R^{+}$ in the two arms is not independent of the overall survival time $T\in R^{+}$ (secondary endpoint).

The observed times can be categorized into the following event types (specified in DepCevent): The event of interest (death from any cause corresponding to overall survival), loss of follow-up (potentially dependent censoring), and end of follow-up period (administrative/independent censoring).

**Table:** 

	5-FU	5-FU + Oxaliplatin
Administrative censoring	469	466
Event of interest	106	96
Loss of follow-up	48	51

The model accommodating dependent censoring can also be fitted using the Coxph() function by appropriately specifying the event in the “Surv” object.


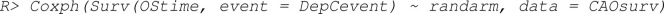


**Table:** 

Coefficient	Estimate	Std. Error	95%-Wald CI
$\beta_{T}$	−0.031	0.143	−0.311 to 0.248
ξ	−0.021	0.452	−0.865 to 0.908
Log-Likelihood			
− 3061.65			

The joint model is fitted based on a Gaussian copula, estimating a marginal flexible proportional hazards model (2) for the overall survival time T and a marginal Weibull proportional hazards model (1) for the loss of follow-up time C , while accounting for independent right-censoring at the end of the follow-up period.

The estimated hazard ratio assessing the treatment effect on overall survival, is $\exp\;({\hat{\beta }}_{T})=0.969$ with a 95%-confidence interval from 0.733 to 1.282 . This indicates no evidence for prolonged overall survival in the experimental compared to the control arm. The estimated dependence parameter is $\hat{\xi }=0.021$ , corresponding to a Kendall’s $\hat{\tau }=-0.014$ . The corresponding 95%-confidence interval from − 0.865 to 0.908 for ξ does include zero, providing no evidence for a dependence between time of overall survival T and loss of follow-up C given the treatment assignment W=w .

## 5. Dependent observations

### 5.1. Survival models

Up to this point, the models we have discussed have been built upon the assumption of independent observations. However, this assumption may not hold in situations where observations are clustered, such as for multi-center trials where observations from the same center are generally correlated.

#### 5.1.1. Mixed-effects proportional hazards model

In order to address this challenge, we can leverage a flexible mixed-effects proportional hazards model as proposed by Tamási et al.^28^ This approach extends the previously discussed smooth transformation models by conditioning on an unobserved cluster-specific random effect R=r that accounts for the dependence within clusters,

$$S_{w}(t|R=r)=P(T>t|W=w,R=r)=\exp\;\left\{-\exp\;\left[h(t)+\beta w+r\right]\right\}.$$

This formulation provides a fully parametric version of the Cox proportional hazards model (2), incorporating multivariate normally distributed random effects with a zero mean and variance $\tau^{2}$ . The treatment effect β is interpreted as a log-hazard ratio conditional on unobserved random effects. For more in-depth information on likelihood-based inference, see Tamási and Hothorn^29^ and Tamási et al.^28^

#### 5.1.2. Marginalized proportional hazards model

Furthermore, we can explore the model proposed by Barbanti and Hothorn,^30^ where the marginal survivor functions are characterized by models (2), while the correlations within clusters are captured by a joint multivariate normal distribution. This joint modeling approach yields a marginalized formulation for the random intercept model,

$$S_{w}(t)=P(T>t|W=w)=\exp\;\left\{-\exp\;\left[\frac{h(t)+\beta w}{\sqrt{\xi^{2}+1}}\right]\right\}.$$

Here, $\xi^{2}$ represents the variance of a cluster-specific intercept. Using this framework, it becomes possible to quantify the treatment effect using the marginal hazard ratio given by $\exp\;(\frac{\beta }{\sqrt{\xi^{2}+1}})$ .

Further details on the models, including likelihood-based inference, can be found in Barbanti and Hothorn.^30^

### 5.2. Application

To estimate mixed-effects smooth transformation models (Section 5.1.1) we can use the **tramME** package,^7,29^ available from CRAN:


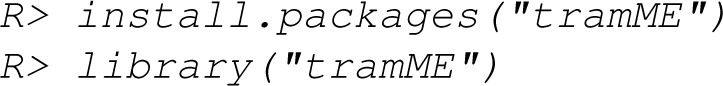


Including a random-intercept for the block used in the randomization, which is a combination of the centers j=1,…88 and the stratum s ( j×s=Block ) in the model, we can account for potential correlation between patients from the same block. The corresponding mixed-effects proportional hazards model can be estimated using the CoxphME() function.


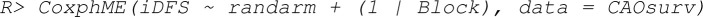


**Table:** 

Coefficient	Estimate	Std. Error	95%-Wald CI
β	−0.235	0.107	−0.445 to −0.026
$\tau^{2}$	0.071		
Log-Likelihood			
− 2241.99			

**Figure 7.**
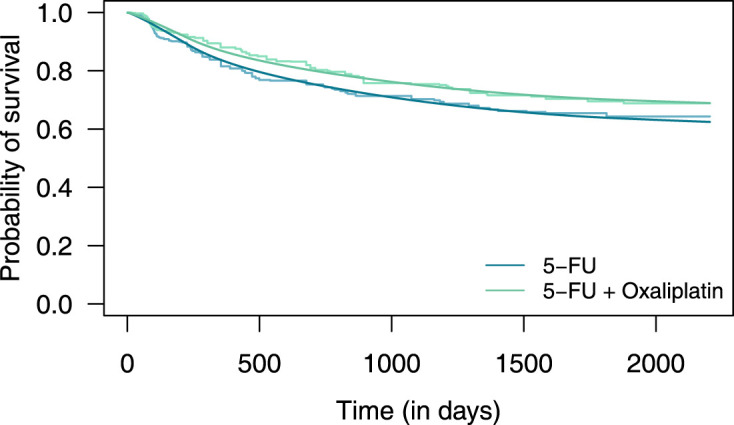
Mixed-effects proportional hazards model. Marginal survivor curves obtained from integrating out the random effects from the model, along with the non-parametric Turnbull estimates.

The model provides an estimate of the log-hazard ratio β , which is conditional on the unobserved random effects. The estimated hazard ratio of $\exp\;(\hat{\beta })=0.790$ and corresponding 95%-confidence intervals indicate prolonged disease-free survival time in the experimental arm. The estimated variance of the random effect R is relatively small, with ${\hat{\tau }}^{2}=0.071$ . We can further examine the corresponding marginal estimates of the survivor curves or related measures by integrating out the random effects (for more details, see Tamási and Hothorn^29^). The corresponding marginal survivor curves for patients from all blocks are depicted in Figure 7.

The estimated mixed-effects proportional hazards model using CoxphME() translates the analysis conducted in Rödel et al.^9^ into the smooth transformation model framework. The aim of the primary analysis of Rödel et al.^9^ was to fit a Cox model for clustered observations estimating the treatment effect and corresponding standard errors. However, at the time of the primary analysis it was not feasible to estimate the mixed-effects Cox model while accounting for interval-censoring. Fortunately, here the discrepancies between the reported results from the model ignoring interval-censoring and the fitted one, accounting for it, are practically negligible.

To obtain a marginal hazard ratio we can estimate the model that captures the dependence within clusters using a joint multivariate normal distribution (Section 5.1.2), which can be fitted using the mtram() function from the **tram** package. Estimation is straightforward for completely exact or interval-censored outcomes within a cluster. Since iDFS comprises a mix of different types of outcomes (within each cluster, event times can be either all exact or all censored, see Formulae 2.5 and 2.6 of Barbanti and Hothorn^30^), we handle exact event times by treating them as interval-censored, accomplished by adding a 4-day window (stored in the object iDFS2 of class “Surv,” see Section 5 of the mtram package vignette^31^ for details).


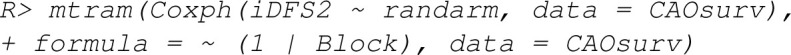


**Table:** 

Coefficient	Estimate	Std. Error	95%-Wald CI
β	−0.235	0.107	−0.446 to −0.025
ξ	0.182	0.117	−0.047 to −0.411
Log-Likelihood			
− 2047.82			

The corresponding estimate of the marginal hazard ratio is $\exp\;(\frac{\hat{\beta }}{\sqrt{{\hat{\xi }}^{2}+1}})=0.794$ with empirical 95%-confidence intervals from 0.647 to 0.978 . The results indicate that the hazards for patients in the experimental arm is reduced by 0.794 compared to the hazards in the control arm, regardless of the block.

## 6. Personalized medicine

In the context of personalized medicine, our focus now turns toward modeling heterogeneous treatment effects to capture a more individualized response to treatment. By fitting models with log-hazard ratios that vary with age, we move beyond a global treatment effect, to assess differences in treatment response across age groups.

### 6.1. Survival models

#### 6.1.1. Age-varying hazards model

To detect varying treatment effects based on age we can employ models, which estimate an age-varying hazard ratio exp(β(age)) ,^6^

$$S_{w}(t)=P(T>t|W=w,\text{Age}=\text{age})=\exp\;\left\{-\exp\;\left[h(t)+\beta (\text{age})w\right]\right\}.$$

This formulation aligns with the model estimated in the analysis of Hofheinz et al.^12^

Such models could be further extended to additionally capture variations in baseline risks across age by including an age-dependent log-cumulative baseline hazard function: $\log\;(\Lambda_{0}(t|\text{age}))=h(t|\text{age})=a{\left(t\right)}^{⊤}\vartheta +\beta_{0}(\text{age})$ .

#### 6.1.2. Tree-based age-varying hazards model

Furthermore, for estimating heterogeneous treatment effects, tree-based Cox models can also be employed,^32,33^

$$S_{w}(t|\text{Age}=\text{age})=\exp\;\left\{-\exp\;\left[h(t|\text{age})+\beta (\text{age})w\right]\right\},$$

allowing to partition both the log-cumulative baseline hazard $\log\;(\Lambda_{0}(t|\text{age}))=h(t|\text{age})=a{\left(t\right)}^{⊤}\vartheta (\text{age})$ and the treatment effect β(age) with respect to different age groups. In contrast to the model in Section 6.1.1, here both the log-cumulative baseline hazard and the log-hazard ratio β depend on age, in this case via an age-structured tree.

### 6.2. Application

The hazards model featuring an age-varying treatment effect (Section 6.1.1) can be fitted using the **tramME** package.^7^


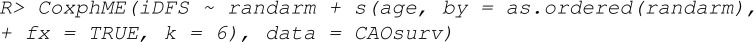


The model estimates a global treatment effect and an additional smooth effect for age in the experimental arm, specified as an unpenalized term (using fx = TRUE) to match the approach used in Hofheinz et al.^12^ From the model terms, one can compute an age-varying hazard ratio exp(β(age)) .

**Figure 8.**
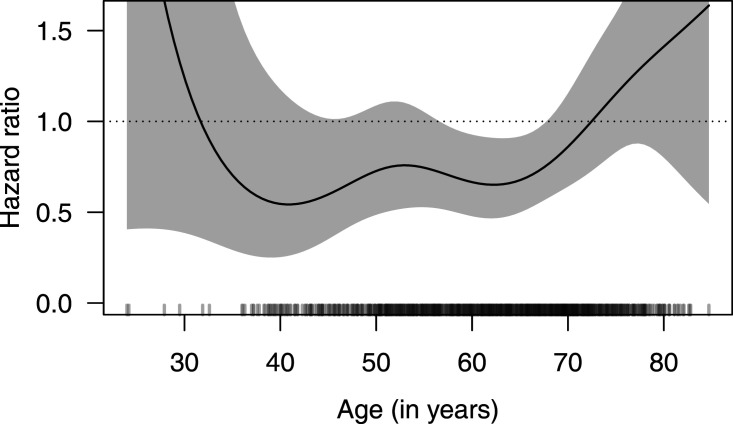
Age-varying hazards model. Hazard ratio and corresponding 95%-confidence interval estimated by the model shown along age. The log-likelihood of the corresponding model is − 2237.63.

The estimated age-varying hazard ratio curve, shown in Figure 8, indicates that the experimental treatment is more effective than the control treatment for patients aged 40−70 years, while for older patients the control treatment reduces the hazard compared to the experimental treatment. The corresponding 95%-confidence interval, however, is notably wide and mostly overlaps with a hazard ratio of 1.

Fitting a model partitioning the log-cumulative baseline hazards and treatment effect by age, a survival tree (Section 6.1.2) can be estimated using the **trtf** package.^8,34^


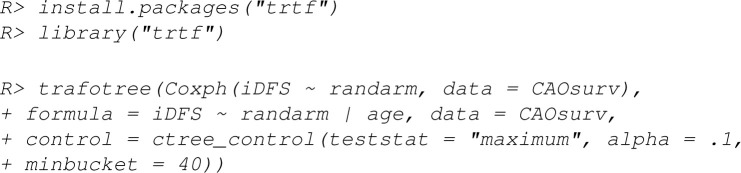


**Figure 9.**
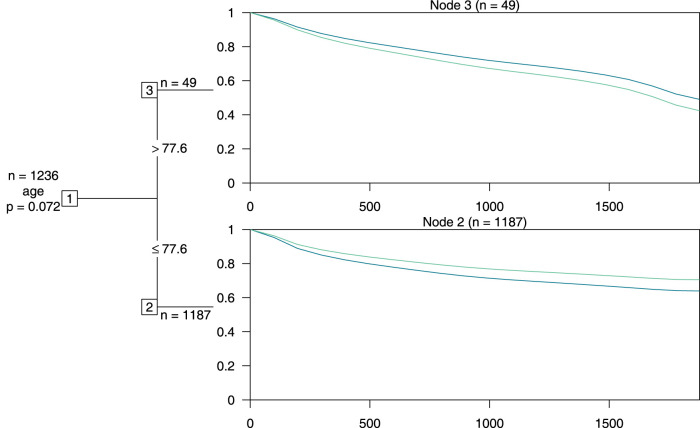
Tree-based age-varying hazards model. Survival tree depicting the estimated survivor curves of the age-groups corresponding to the terminal nodes of the partitioned hazards model. The corresponding in-sample log-likelihood is − 2236.21.

The survivor functions corresponding to the terminal nodes of the estimated tree are shown in Figure 9. The results again indicate that the experimental treatment is more effective for younger patients, while the control treatment is more effective for older patients. This result is also in line with the one previously obtained by Hofheinz et al.^12^

## 7. Other extensions

### 7.1. Frailty proportional hazards model

In cases where the assumption of a homogeneous study population falls short, frailty models offer a valuable alternative. These models account for unobserved heterogeneity in scenarios where the study population comprises individuals with varying baseline risks.^35^

To handle such scenarios in the framework of smooth transformation models, the approach discussed by McLain and Ghosh^16^ can be employed. The corresponding frailty proportional hazards model introduces an unobservable multiplicative frailty effect A on the hazard, with corresponding conditional survivor function

$$S_{w}(t|A=a)=P(T>t|W=w,A=a)=\exp\;\left\{-\exp\;\left[h(t)+\log\;(a)+\beta w\right]\right\}.$$

The model implies that the proportional hazards assumption, $\frac{\Lambda_{1}(t|a)}{\Lambda_{0}(t|a)}=\frac{\lambda_{1}(t|a)}{\lambda_{0}(t|a)}=\beta$ , holds conditional on frailty A=a . The frailty A specifies a latent random term, assumed to have a certain non-negative distribution, such as the gamma, inverse Gaussian or positive stable distribution,^36^ which, for identifiability, is scaled such that E(A)=1 . The proportional hazards model with gamma frailty can be fitted in **tram**, using the Coxph() function specifying the frailty distribution with frailty = "Gamma".

### 7.2. Non-parametric likelihood

In this tutorial, we have primarily focused on the implementation of smooth parameterization for the log-cumulative baseline hazard function h . Nevertheless, it is important to highlight that researchers also have the option to utilize the discussed models that incorporate a non-parametric version of the transformation function h in package **tram**, should they wish to do so. The corresponding non-parametric transformation function h is specified in terms of $a(t_{k}{)}^{⊤}\vartheta =\vartheta_{k}$ , where a parameter $\vartheta_{k}$ is assigned to each distinct event time $t_{k}$ with k=1,…,K−1 . A head-to-head comparison of the smooth parametrization and the non-parametric version can be found in Tian et al.^19^

### 7.3. Link function

Undoubtedly, the proportional hazards model stands as a cornerstone in survival analysis, prominently emerging from specifying the complementary log-log link (the cumulative distribution function of the standard minimum extreme value distribution), wherein h parameterizes the log-cumulative baseline hazard function. Nevertheless, it is worth noting that researchers have the option to explore other link functions for all the models shown above, such as the logit link (as also discussed in detail in Royston and Parmar^37^), or the probit or log-log link.

For example, specifying a flexible proportional odds model “only” requires to change the link function from complementary log-log to logit; such a model can be estimated via


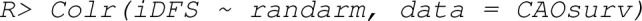


**Table:** 

Coefficient	Estimate	Std. Error	95%-Score CI
β	−0.291	0.124	−0.534 to −0.047
Log-Likelihood	Likelihood Ratio Test	Score Test	Permutation Score Test
− 2242.06	p = 0.019	p = 0.019	p = 0.019

This inherent versatility of link functions facilitates to construct alternative models, including mean or odds models, by specifying a probit or logit link respectively. These models are well known from the class of accelerated failure time models (with log-linear h ), but extend seamlessly to the more flexible framework of smooth transformation models. Moreover, by selecting the log-log link, it is possible to define a reverse time hazards model. For a comprehensive overview, see Table 1 of Hothorn et al.^3^

### 7.4. Covariate adjustment

While the models above focus on estimating the treatment effect, they can naturally extend to incorporate further covariates x . For example, in the time-varying hazards model (Section 3.1.5), age can be incorporated into the linear predictor as follows:


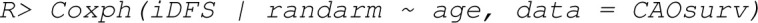


Additionally, penalized covariate effects can be estimated by maximizing the $L_{1}$ - or $L_{2}$ -penalized log-likelihood using the **tramnet** package.^38,39^

Moreover, conditional transformation models,^40^ which accommodate complex, non-linear covariate effects, can be estimated using package **tbm**.^41,42^

### 7.5. Sample size estimation and simulation

The framework of smooth transformation models can also be valuable for researchers involved in designing new studies. Simulating from the illustrated models (using simulate()) offers a straightforward approach for tasks such as sample size estimation. Because the transformation function h is smooth, it is relatively simple to invert this function numerically, such that it becomes possible to apply probability integral transforms for generating new event times from a fitted model analogously to Bender et al.^43^ As an example, we might want to generate data for a future trial where 5-FU overall survival is improved by some innovative therapy. We start with fitting a Weibull model to overall survival, conditional on treatment w and age.


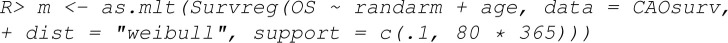


We simulate new survival times T from this conditional distribution for study participants with normally distributed age in a balanced trial, with the covariate values stored in a data frame called nd. A useful feature in **tram** is the ability to change model coefficients on the fly. Here, we change the log-hazard ratio to 0.25 and simulate from this altered Weibull model:


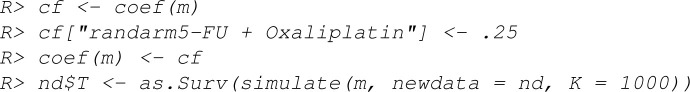


In addition, we simulate censoring times C⊥⊥T∣W=w,Age=age such that 80% of observations will be right-censored (with probabilistic index $P(T>C|W=w,\text{Age}=\text{age})=0.8={\mathrm{logit}}^{-1}\;(1.386)$ ^44^)


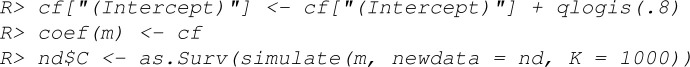


and finally compute the potentially right-censored event times for model re-fitting:


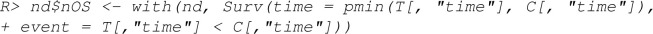


## 8. Discussion

Motivated by the complexities researchers face when navigating various software implementations for survival analysis, we outline the potential of leveraging smooth transformation models^3^ in R. Together with related add-on packages such as **tramME**^7^ and **trtf**,^8^ the **tram** package provides a unified maximum likelihood framework that seamlessly extends classical survival models to accommodate more complex scenarios, offering a versatile toolkit for survival analysis.

Throughout this tutorial, we present practical strategies for addressing prominent challenges in survival analysis in R, including interval-censored outcomes, non-proportional or crossing hazards, dependent censoring, clustered observations, and heterogeneous treatment effects. The comparative overview of implementations in Supplementary Material A serves as a validation tool, allowing researchers to compare across multiple packages.

The framework’s modular architecture further allows individual model components to be combined—for example, covariate-dependent hazard ratios can be paired with random effects using **tramME**. The framework also extends to multiple event times per subject, allowing for the estimation of multivariate survival models via the Mmlt() function.^45^ This flexibility provides users with a unified toolkit to seamlessly address a wide spectrum of complex survival analysis tasks in R.

The implemented framework also provides the foundation for interesting extensions. For example, the model in Section 3.1.4 could be adapted to handle cured patients, as a fully parametric version of the semi-parametric cure mixture model proposed by Xie et al.^46^ In the context of covariate adjustment, extending the implementation to collapsible models similar to Crowther et al.^47^ could be explored. Additionally, alternative strategies such as marginalizing the hazard ratio, as suggested by Daniel et al.,^48^ could also be explored further.

## Supplemental Material

sj-pdf-1-smm-10.1177_09622802251414595 - Supplemental material for Smooth transformation models for survival analysis: A tutorial using R Supplemental material, sj-pdf-1-smm-10.1177_09622802251414595 for Smooth transformation models for survival analysis: A tutorial using R by Sandra Siegfried, Bálint Tamási and Torsten Hothorn in Statistical Methods in Medical Research
