# Supplementary material for: Smooth transformation models for survival analysis: A tutorial using R
Source: Stat Methods Med Res. 2026 Apr 27;35(5):980–97. doi: 10.1177/09622802251414595 (PMC13272841; doi:10.1177/09622802251414595)
Supplement: sj-pdf-1-smm-10.1177_09622802251414595 - Supplemental material for Smooth transformation models for survival analysis: A tutorial using R [file sj-pdf-1-smm-10.1177_09622802251414595.pdf]

Supplementary Material for "Smooth Transformation Models for Survival Analysis: A Tutorial Using R"

Sandra Siegfried
Universität Zürich

Bálint Tamási
Universität Zürich

Torsten Hothorn
Universität Zürich

A. Comparative overview of R implementations

In the following, we compare the implementations of the models from the **tram** (Hothorn *et al.* 2025) and **tramME** (Tamási 2025b) packages shown in the tutorial, with alternative models available in various established R packages from CRAN.

We fit the models to the primary endpoint of disease-free survival, which comprises a mixture of exact times and right- and interval-censored event times (encoded in `iDFS`, an object of class '`Surv`'). In order to further compare the models with other implementations that cannot handle interval-censored outcomes, we treat the interval-censored observations as if they were observed exactly (encoded in `DFS`, an object of class '`Surv`').

We contrast treatment effect estimates (Estimate) and corresponding standard errors (Std. Error) estimated by the fitted models. It is important to note the difference in interpretation of the estimates (Interpretation). Additionally, we provide the in-sample log-likelihood (logLik) of the fitted models, with penalised or semi-parametric/partial likelihoods highlighted in grey.

A.1. Weibull models

The **survival** package (Therneau 2024b), the **icenReg** package (Anderson-Bergman 2024, 2017) and the **flexsurv** package (Jackson 2024) provide alternative implementations of Weibull models. Both the **survival** and **icenReg** package (specifying `model = "aft"`) implement accelerated failure time Weibull models, where the effect can be interpreted as log-acceleration factor (log-AF). An alternative parametrisation of such models is in terms of proportional hazards Weibull models, estimating log-hazard ratios (log-HRs), instead. This is how the model, fitted by `Survreg()`, is implemented in the **tram** package (Hothorn *et al.* 2025), which can be directly compared to the analogous parametrisation in the **icenReg** (specifying `model = "ph"`) and the **flexsurv** package (with `dist = "weibullPH"`). All Weibull models can handle interval-censoring, owing to the parametric nature of the models.

The Weibull models can be fitted to the *interval-censored outcomes* as follows:

```
R> tram::Survreg(iDFS ~ randarm, data = CAOsurv, dist = "weibull")
R> icenReg::ic_par(iDFS ~ randarm, data = CAOsurv, dist = "weibull",
+    model = "ph")
R> flexsurv::flexsurvreg(iDFS ~ randarm, data = CAOsurv,
+    dist = "weibullPH")
```

```
R> survival::survreg(iDFS ~ randarm, data = CAOsurv, dist = "weibull")
R> icenReg::ic_par(iDFS ~ randarm, data = CAOsurv, dist = "weibull",
+    model = "aft")
```

Function	Package	Interpretation	Estimate	Std. Error	logLik
`Survreg`	**tram**	log-HR	−0.229	0.106	−2’281.17
`ic_par`	**icenReg**	log-HR	−0.229	0.106	−2’281.17
`flexsurvreg`	**flexsurv**	log-HR	−0.229	0.106	−2’281.17
`survreg`	**survival**	log-AF	0.312	0.146	−2’281.17
`ic_par`	**icenReg**	log-AF	0.312	0.146	−2’281.17

As expected, all packages provide equivalent model fits.

A.2. Flexible proportional hazards models

Flexible versions of the proportional hazards model are implemented in several packages, of which the following accommodate interval-censored outcomes. The **rstpm2** package (Clements *et al.* 2025; Liu *et al.* 2016) and the **flexsurv** package provide parametric versions of the model by using splines (analogously to the approach discussed by Royston and Parmar (2002)). We set `k = 3` for the number of knots in the spline for `flexsurvspline()` from the **flexsurv** package Alternatively, the **icenReg** package (Anderson-Bergman 2024) provides a semi-parametric implementation of the model that can handle interval-censoring.

The corresponding models can be fitted to the *interval-censored outcomes* as follows:

```
R> tram::Coxph(iDFS ~ randarm, data = CAOsurv)
R> rstpm2::stpm2(Surv(time = iDFStime, time2 = iDFStime2,
+    event = iDFSevent, type = "interval") ~ randarm, data = CAOsurv)
R> flexsurv::flexsurvspline(iDFS ~ randarm, data = CAOsurv, k = 3)
R> icenReg::ic_sp(iDFS ~ randarm, data = CAOsurv, model = "ph")
```

Function	Package	Interpretation	Estimate	Std. Error	logLik
`Coxph`	**tram**	log-HR	−0.231	0.107	−2’242.25
`stpm2`	**rstpm2**	log-HR	−0.232	0.107	−2’250.48
`flexsurvspline`	**flexsurv**	log-HR	−0.231	0.106	−2’254.34
`ic_sp`	**icenReg**	log-HR	−0.230	–	−1’977.29

The models fit similarly across all four packages. Due to the fact that the computations of the standard errors of `ic_sp()` from the **icenReg** package rely on computationally expensive bootstrap sampling, we did not report any standard errors for this approach. Also the log-likelihood (in grey) of the semi-parametric model from the **icenReg** is not comparable to the otherwise fully parametric implementations.

The **ICsurv** package (McMahan and Wang 2022) could also potentially handle interval-censored event times. The **TransModel** package (Zhou *et al.* 2022b,a), featuring an alternative implementation of linear transformation model, could also serve as an interesting comparator. However, we encountered difficulties and errors when trying to fit the model using these two packages.

In scenarios where *interval-censoring* is not taken into account, there are several other implementations available for fitting corresponding models. The `coxph()` function from the **survival**

package provides a semi-parametric approach for exact or right-censored observations (Therneau 2024b). (Note, that again the likelihood of the fitted model is not comparable to the other fully parametric models and thus marked in grey). The **rms** package (Harrell Jr 2025) implements a semi-parametric model, in line with the model from package **survival**.

```
R> tram::Coxph(DFS ~ randarm, data = CAOsurv)
R> survival::coxph(DFS ~ randarm, data = CAOsurv)
R> rms::cph(DFS ~ randarm, data = CAOsurv)
```

Function	Package	Interpretation	Estimate	Std. Error	logLik
Coxph	**tram**	log-HR	−0.230	0.106	−3'264.89
coxph	**survival**	log-HR	−0.228	0.106	−2'430.66
cph	**rms**	log-HR	−0.228	0.106	−2'430.66

A.3. Stratified proportional hazards models

For comparing stratified flexible proportional hazards models we can again utilize the model from the **rstpm2**, which employ stratified spline-basis functions. The model can be fitted to the *interval-censored event times* as follows

```
R> tram::Coxph(iDFS | strat ~ randarm, data = CAOsurv)
R> rstpm2::stpm2(Surv(time = iDFStime, time2 = iDFStime2,
+       event = iDFSevent, type = "interval") ~ randarm +
+       strata(strat), data = CAOsurv)
```

Function	Package	Interpretation	Estimate	Std. Error	logLik
Coxph	**tram**	log-HR	−0.228	0.107	−2'213.94
stpm2	**rstpm2**	log-HR	−0.220	0.107	−2'242.88

The results from the two models are practically similar.

Now, *ignoring interval-censoring*, we can, once again, contrast the implementation of the semi-parametric models from the **survival** package and the **rms** package:

```
R> tram::Coxph(DFS | strat ~ randarm, data = CAOsurv)
R> survival::coxph(DFS ~ randarm + strata(strat), data = CAOsurv)
R> rms::cph(DFS ~ randarm + strat(strat), data = CAOsurv)
```

Function	Package	Interpretation	Estimate	Std. Error	logLik
Coxph	**tram**	log-HR	−0.228	0.107	−3'234.58
coxph	**survival**	log-HR	−0.222	0.107	−2'089.54
cph	**rms**	log-HR	−0.222	0.107	−2'089.54

The three model fits are practically equivalent.

We can proceed to compare the stratified version of the Weibull model, for which we also will ignore interval-censoring due to the fact that the utilised **eha** package (Broström 2024) lacks support for interval-censored data. Additionally, it is worth highlighting that there is

a distinction from the model fitted using `survreg()` from the **survival** package (Therneau 2024b). This model only stratifies the scale parameter of the Weibull distribution, whereas the models from the **eha** package and the **tram** package estimate both strata-dependent scale and shape terms. The `survreg` function from the **survival** package fits an accelerated failure time Weibull model, while the **eha** package implements a proportional hazards Weibull model, analogously to the `Survreg()` implementation from the **tram** package. The models can be fitted to the exact event times, *ignoring interval-censoring*, as follows

```
R> tram::Survreg(DFS | strat ~ randarm, data = CAOsurv)
R> eha::phreg(DFS ~ randarm + strata(strat), data = CAOsurv)
R> survival::survreg(DFS ~ randarm + strata(strat), data = CAOsurv)
```

Function	Package	Interpretation	Estimate	Std. Error	logLik
`Survreg`	**tram**	log-HR	−0.219	0.107	−3’277.35
`phreg`	**eha**	log-HR	−0.219	0.107	−3’277.35
`survreg`	**survival**	log-AF	0.274	0.133	−3’280.87

The fit of the `survreg` model from **survival** package is slightly different. In contrast, the parametrisation and fits of the model from the **eha** and the **tram** are equivalent.

A.4. Non-proportional hazards models

To the best of our knowledge, there is currently no implementation available that estimates an analogous model to the flexible non-proportional (location-scale) hazards model implemented in **tram**.

However we can contrast the implementation of the less-flexible Weibull model with the **gamlss** package (Stasinopoulos and Rigby 2025, 2007) using the `WEI2` distribution and the **gamlss.cens** package (Stasinopoulos *et al.* 2023) to account for *interval-censoring*.

```
R> tram::Survreg(iDFS ~ randarm | randarm, data = CAOsurv,
+    remove_intercept = FALSE)
R> gamlss::gamlss(formula = iDFS ~ randarm, sigma.fo = ~ randarm,
+    family = gamlss.cens::cens(family = "WEI2", type = "interval"),
+    data = CAOsurv[, c("iDFS", "randarm")],
+    control = gamlss.control(n.cyc = 300, trace = FALSE))
```

Since the scale term in the **tram** package and the **gamlss** package are parameterised differently, we only show the estimates and standard errors of the location parameter below.

Function	Package	Estimate	Std. Error	logLik
`Survreg`	**tram**	−0.847	0.536	−2’280.47
`gamlss`	**gamlss**	−0.948	0.542	−2’280.53

The two implementations provide almost equivalent model fits.

The **mpr** package (Burke 2022) also offers an implementation for a non-proportional Weibull model, it, however, does not support interval-censored data. Thus we fit the models *ignoring interval-censoring*.

```
R> tram::Survreg(DFS ~ randarm | randarm, data = CAOsurv,
+    remove_intercept = FALSE)
R> mpr::mpr(DFS ~ list(~ randarm, ~ randarm), data = CAOsurv)
```

Again, we only show the estimates and standard errors of the location parameter below.

Function	Package	Estimate	Std. Error	logLik
`Survreg`	**tram**	−0.976	0.568	−3'290.43
`mpr`	**mpr**	−0.975	0.567	−3'290.43

The two implementations also provide equivalent model fits.

A.5. Time-varying hazards model

We can compare the time-varying hazards model from the **tram** and the **flexsurv** package (Jackson 2024) which allows to estimate time-varying treatment effects. We start by examining the models for the *interval-censored event times*.

```
R> tram::Coxph(iDFS | randarm ~ 1, data = CAOsurv)
R> flexsurv::flexsurvspline(iDFS ~ randarm + gamma1(randarm) +
+      gamma2(randarm), data = CAOsurv, k = 3)
```

The in-sample log-likelihood is −2'252.95 for the **flexsurv** model and −2'240.21 for the **tram** model. The estimated time-varying ratios of the cumulative hazards are shown in the plot below.

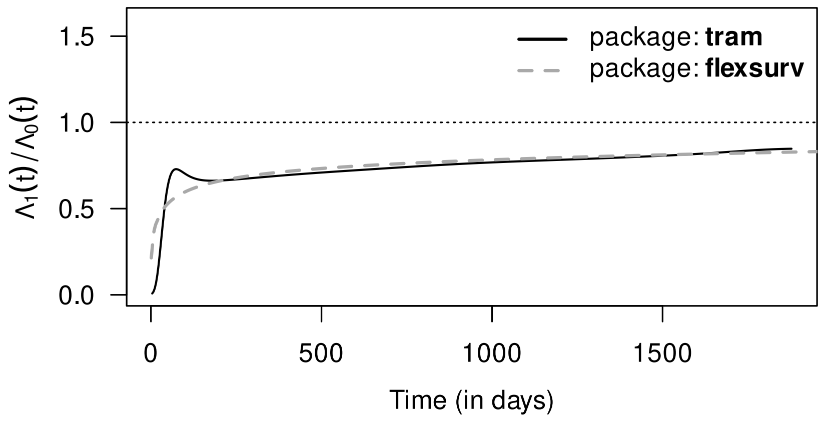

We will now explore the same models *ignoring interval-censoring*.

```
R> tram::Coxph(DFS | randarm ~ 1, data = CAOsurv)
R> flexsurv::flexsurvspline(DFS ~ randarm + gamma1(randarm) +
+      gamma2(randarm), data = CAOsurv, k = 3)
```

The in-sample log-likelihood is −3'267.27 for the **flexsurv** model and −3'262.54 for the **tram** model, with the computed time-varying ratios of the cumulative hazards shown below.

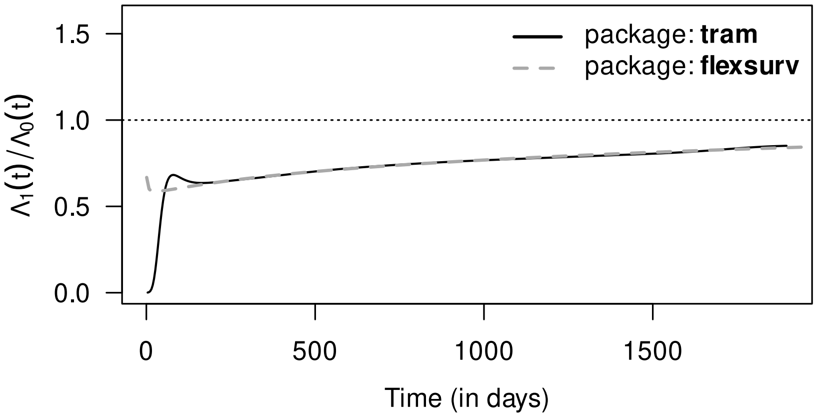

The time-varying effects estimated from `DFS` show good agreement, the ratios slightly differ when the models are estimated on the interval-censored data (`iDFS`).

A.6. Mixed-effects proportional hazards models

The implementation of a mixed-effects proportional hazards model with flexible log-cumulative baseline hazards for interval-censored event times is unique in the **tramME** package (Tamási 2025b; Tamási and Hothorn 2021). While the **rstpm2** package also accommodates interval-censored event times, we were not able to fit the corresponding mixed-effects model to our data.

Thus, to contrast the models with other implementations we, again, need to *ignore interval-censoring*. We can then compare the fitted model with the fully parametric spline-based version from the **rstpm2** package (Clements *et al.* 2025; Liu *et al.* 2017) and the semi-parametric model estimated by the **coxme** package (Therneau 2024a), employing Gaussian random effects using a Laplace approximation (Ripatti and Palmgren 2000).

```
R> tramME::CoxphME(DFS ~ randarm + (1 | Block), data = CAOsurv)
R> rstpm2::stpm2(Surv(DFStime, DFSevent) ~ randarm, data = CAOsurv,
+    cluster = "Block", RandDist = "LogN")
R> coxme::coxme(DFS ~ randarm + (1 | Block), data = CAOsurv)
```

Function	Package	Interpretation	Estimate	Std. Error	logLik
`CoxphME`	**tramME**	log-HR	−0.234	0.107	−3'264.66
`stpm2`	**rstpm2**	log-HR	−0.234	0.107	−3'272.86
`coxme`	**coxme**	log-HR	−0.231	0.107	−2'414.48

The fitted models from the three packages agree very well.

A.7. Age-varying hazards model

We can compare the age-varying hazards model from package **tramME** (Tamási 2025a) to the implementation in the **mgcv** package (Wood 2025; Wood *et al.* 2016) which estimates a smooth Cox model via partial likelihood optimisation. As the model from the **mgcv** package only accommodates right-censored observations we again fit the models *ignoring interval-censoring*.

```
R> tramME::CoxphME(DFS ~ randarm + s(age, by = as.ordered(randarm),
+       fx = TRUE, k = 6), data = CAOsurv)
R> mgcv::gam(DFStime ~ randarm + s(age, by = as.ordered(randarm),
+       fx = TRUE, k = 6), data = CAOsurv, family = cox.ph(),
+    weights = DFSevent)
```

The in-sample log-likelihood of the model from the package **mgcv** is −2'426.04 (partial log-likelihood) and −3'260.25 for the **tramME** model. The estimated age-varying hazard ratios and corresponding 95%-confidence intervals are shown in the plot below.

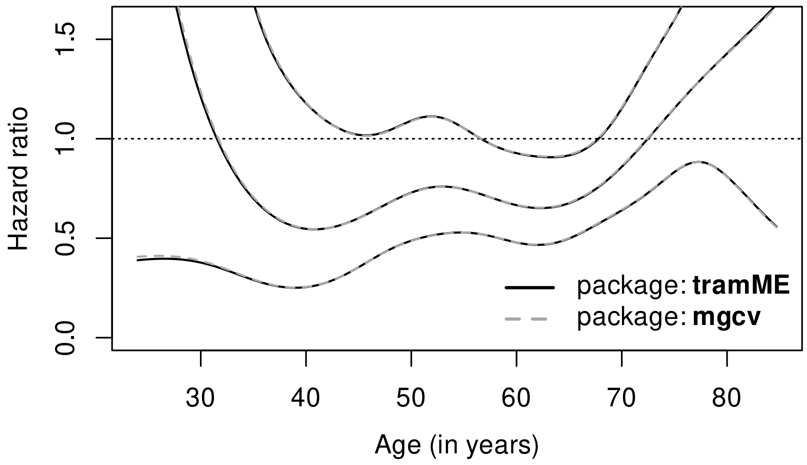

The hazard ratio curves and confidence intervals estimated by the two packages are practically equivalent.

A.8. Frailty proportional hazards models

For models featuring a gamma frailty, we can contrast implementations using a semi-parametric approach or the spline-based approach from the **rstpm2** package (Liu *et al.* 2017). The `coxph()` model from the **survival** package uses a semi-parametric approach and estimates the frailty term using penalised regression (Therneau *et al.* 2003). The **frailtyEM** (Balan and Putter 2019b,a) and the **frailtypack** package (Rondeau *et al.* 2025, 2012) also feature models with semi-parametric baseline hazards. Again we fit the models *ignoring interval-censoring.*

```
R> tram::Coxph(DFS ~ randarm, data = CAOsurv, frailty = "Gamma")
R> rstpm2::stpm2(Surv(DFStime, DFSevent) ~ randarm, data = CAOsurv,
+    cluster = "id", RandDist = "Gamma")
R> survival::coxph(DFS ~ randarm + frailty(id, distribution = "gamma"),
+    data = CAOsurv)
R> frailtyEM::emfrail(DFS ~ randarm + cluster(id), data = CAOsurv)
R> frailtypack::frailtyPenal(DFS ~ randarm + cluster(id),
+    data = CAOsurv, RandDist = "Gamma", n.knots = 10, kappa = 1)
```

Function	Package	Interpretation	Estimate	Std. Error	logLik
`Coxph`	**tram**	log-HR	−0.230	0.107	−3'264.89
`stpm2`	**rstpm2**	log-HR	−0.685	0.670	−3'264.88
`coxph`	**survival**	log-HR	−0.406	0.159	−1'944.22
`emfrail`	**frailtyEM**	log-HR	−0.384	0.153	−2'430.45
`frailtyPenal`	**frailtypack**	log-HR	−0.660	0.248	−3'259.82

The fitted models vary considerably across packages.

A.9. Flexible proportional odds models

We can compare the fit of the flexible proportional odds model with the implementation in the **rstpm2** package (Clements *et al.* 2025) and package **flexsurv** (Jackson 2024). The `Gprop.odds` function from package **timereg** (Scheike and Martinussen 2025; Scheike and Zhang 2011) can also estimate a flexible proportional odds model using the partial likelihood, thus we again compare the models *ignoring interval-censoring.*

```
R> tram::Colr(DFS ~ randarm, data = CAOsurv)
R> rstpm2::stpm2(Surv(DFStime, DFSevent) ~ randarm, data = CAOsurv,
+    link.type = "PO")
R> flexsurv::flexsurvspline(iDFS ~ randarm, data = CAOsurv, k = 3,
+    scale = "odds")
R> timereg::Gprop.odds(DFS ~ prop(randarm), data = CAOsurv)
```

Function	Package	Interpretation	Estimate	Std. Error	logLik
`Colr`	**tram**	log-OR	−0.292	0.125	−3'265.48
`stpm2`	**rstpm2**	log-OR	−0.294	0.125	−3'272.44
`flexsurvspline`	**flexsurv**	log-OR	−0.294	0.124	−2'247.78
`Gprop.odds`	**timereg**	log-OR	−0.268	0.125	

The fitted models are practically equivalent among the four packages. Note, that we were not able to retrieve the in-sample log-likelihood from the model object of the **timereg** package and thus do not report it here.

Computational details

- R version 4.5.2 (2025-10-31), `x86_64-pc-linux-gnu`
- Running under: `Ubuntu 24.04.3 LTS`
- Matrix products: default
- BLAS: `/usr/lib/x86_64-linux-gnu/openblas-pthread/libblas.so.3`
- LAPACK: `/usr/lib/x86_64-linux-gnu/openblas-pthread/libopenblasp-r0.3.26.so`; LAPACK version3.12.0
- Base packages: `base`, `datasets`, `graphics`, `grDevices`, `grid`, `methods`, `parallel`, `splines`, `stats`, `utils`

- Other packages: `ATR 0.1-1, basefun 1.2-5, bdsmatrix 1.3-7, boot 1.3-32, coda 0.19-4.1, coin 1.4-3, colorspace 2.1-2, coxme 2.2-22, doBy 4.7.0, eha 2.11.5, fastGHQuad 1.0.1, flexsurv 2.3.2, frailtyEM 1.0.1, frailtypack 3.7.1, gamlss 5.5-0, gamlss.cens 5.0-7, gamlss.data 6.0-7, gamlss.dist 6.1-1, Hmisc 5.2-4, icenReg 2.0.16, ICsurv 1.0.1, knitr 1.50, libcoin 1.0-10, MASS 7.3-65, mgcv 1.9-4, mlt 1.7-2, mpr 1.0.6, multcomp 1.4-29, mvtnorm 1.3-3, nlme 3.1-168, optimx 2025-4.9, parfm 2.7.8, partykit 1.2-24, Rcpp 1.1.0, rms 8.1-0, rstpm2 1.7.1, SparseGrid 0.8.2, survC1 1.0-3, survival 3.8-3, TH.data 1.1-5, timereg 2.0.7, tram 1.3-0, tramME 1.0.8, TransModel 2.3, trtf 0.4-3, variables 1.1-2, xtable 1.8-4`

- Loaded via a namespace (and not attached): `alabama 2023.1.0, assertthat 0.2.1, backports 1.5.0, base64enc 0.1-3, BB 2019.10-1, bbmle 1.0.25.1, broom 1.0.10, checkmate 2.3.3, cli 3.6.5, cluster 2.1.8.1, codetools 0.2-20, compiler 4.5.2, coneproj 1.22, cowplot 1.2.0, data.table 1.17.8, Deriv 4.2.0, deSolve 1.40, digest 0.6.38, dplyr 1.1.4, evaluate 1.0.5, expint 0.1-9, expm 1.0-0, farver 2.1.2, fastmap 1.2.0, foreach 1.5.2, foreign 0.8-90, Formula 1.2-5, future 1.67.0, future.apply 1.20.0, generics 0.1.4, ggplot2 4.0.1, globals 0.18.0, glue 1.8.0, gridExtra 2.3, gtable 0.3.6, htmlTable 2.4.3, htmltools 0.5.8.1, htmlwidgets 1.6.4, inum 1.0-5, iterators 1.0.14, lattice 0.22-7, lava 1.8.2, lifecycle 1.0.4, listenv 0.10.0, magrittr 2.0.4, Matrix 1.7-4, matrixcalc 1.0-6, MatrixModels 0.5-4, matrixStats 1.5.0, microbenchmark 1.5.0, mnormt 2.1.1, modelr 0.1.11, modeltools 0.2-24, msm 1.8.2, mstate 0.3.3, muhaz 1.2.6.4, nloptr 2.2.1, nnet 7.3-20, numDeriv 2016.8-1.1, orthopolynom 1.0-6.1, parallelly 1.45.1, pillar 1.11.1, pkgconfig 2.0.3, polspline 1.1.25, polynom 1.4-1, pracma 2.4.6, purrr 1.2.0, quadprog 1.5-8, quantreg 6.1, R6 2.6.1, rbibutils 2.4, RColorBrewer 1.1-3, Rdpack 2.6.4, reformulas 0.4.2, rlang 1.1.6, rmarkdown 2.30, rootSolve 1.8.2.4, rpart 4.1.24, rstudioapi 0.17.1, S7 0.2.1, sandwich 3.1-1, scales 1.4.0, sn 2.1.1, SparseM 1.84-2, statmod 1.5.1, stats4 4.5.2, stringi 1.8.7, stringr 1.6.0, tibble 3.3.0, tidyr 1.3.1, tidyselect 1.2.1, TMB 1.9.18, tools 4.5.2, vctrs 0.6.5, withr 3.0.2, xfun 0.54, zoo 1.8-14`

References

Anderson-Bergman C (2017). "**icenReg**: Regression Models for Interval Censored Data in R." *Journal of Statistical Software*, **81**(12), 1–23. doi:10.18637/jss.v081.i12.

Anderson-Bergman C (2024). ***icenReg**: Regression Models for Interval Censored Data*. doi:10.32614/CRAN.package.icenReg. R package version 2.0.16.

Balan TA, Putter H (2019a). "**frailtyEM**: An R Package for Estimating Semiparametric Shared Frailty Models." *Journal of Statistical Software*, **90**(7). doi:10.18637/jss.v090.i07.

Balan TA, Putter H (2019b). ***frailtyEM**: Fitting Frailty Models with the EM Algorithm*. doi:10.32614/CRAN.package.frailtyEM. R package version 1.0.1.

Broström G (2024). ***eha**: Event History Analysis*. doi:10.32614/CRAN.package.eha. R package version 2.11.5.

Burke K (2022). ***mpr**: Multi-Parameter Regression (MPR)*. doi:10.32614/CRAN.package.mpr. R package version 1.0.6.

Clements M, Liu XR, Christoffersen B (2025). ***rstpm2**: Smooth Survival Models, Including Generalized Survival Models*. doi:10.32614/CRAN.package.rstpm2. R package version 1.7.1.

Harrell Jr FE (2025). ***rms**: Regression Modeling Strategies*. doi:10.32614/CRAN.package.rms. R package version 8.1-0.

Hothorn T, Siegfried S, Kook L (2025). ***tram**: Transformation Models*. doi:10.32614/CRAN.package.tram. R package version 1.3-0.

Jackson C (2024). ***flexsurv**: Flexible Parametric Survival and Multi-State Models*. doi:10.32614/CRAN.package.flexsurv. R package version 2.3.2.

Liu XR, Pawitan Y, Clements M (2016). "Parametric and Penalized Generalized Survival Models." *Statistical Methods in Medical Research*, **27**(5), 1531–1546. doi:10.1177/0962280216664760.

Liu XR, Pawitan Y, Clements M (2017). "Generalized Survival Models for Correlated Time-To-Event Data." *Statistics in Medicine*, **36**(29), 4743–4762. doi:10.1002/sim.7451.

McMahan CS, Wang L (2022). ***ICsurv**: Semiparametric Regression Analysis of Interval-Censored Data*. doi:10.32614/CRAN.package.ICsurv. R package version 1.0.1.

Ripatti S, Palmgren J (2000). "Estimation of Multivariate Frailty Models Using Penalized Partial Likelihood." *Biometrics*, **56**(4), 1016–1022. doi:10.1111/j.0006-341X.2000.01016.x.

Rondeau V, Gonzalez JR, Mazroui Y, Mauguen A, Diakite A, Laurent A, Lopez M, Krol A, Sofeu CL, Dumerc J, Rustand D, Chauvet J, Le Coent Q, Pierlot R, Etzkorn L, Dinart D, Orué A, Philipps V (2025). ***frailtypack**: Shared, Joint (Generalized) Frailty Models; Surrogate Endpoints*. doi:10.32614/CRAN.package.frailtypack. R package version 3.7.1.

Rondeau V, Marzroui Y, Gonzalez JR (2012). "**frailtypack**: An R Package for the Analysis of Correlated Survival Data with Frailty Models Using Penalized Likelihood Estimation or Parametrical Estimation." *Journal of Statistical Software*, **47**(4), 1–28. doi:10.18637/jss.v047.i04.

Royston P, Parmar MK (2002). "Flexible Parametric Proportional-Hazards and Proportional-Odds Models for Censored Survival Data, with Application to Prognostic Modelling and Estimation of Treatment Effects." *Statistics in Medicine*, **21**(15), 2175–2197. doi:10.1002/sim.1203.

Scheike T, Martinussen T (2025). ***timereg**: Flexible Regression Models for Survival Data.* doi:10.32614/CRAN.package.timereg. R package version 2.0.7.

Scheike TH, Zhang MJ (2011). "Analyzing Competing Risk Data Using the R **timereg** Package." *Journal of Statistical Software*, **38**(2), 1–15. doi:10.18637/jss.v038.i02.

Stasinopoulos DM, Rigby RA (2007). "Generalized Additive Models for Location Scale and Shape (GAMLSS) in R." *Journal of Statistical Software*, **23**(7), 1–46. doi:10.18637/jss.v023.i07.

Stasinopoulos M, Rigby B, Mortan N, Seipp A (2023). ***gamlss.cens**: Fitting an Interval Response Variable Using **gamlss.family** Distributions.* doi:10.32614/CRAN.package.gamlss.cens. R package version 5.0-7.

Stasinopoulos M, Rigby R (2025). ***gamlss**: Generalized Additive Models for Location Scale and Shape.* doi:10.32614/CRAN.package.gamlss. R package version 5.5-0.

Tamási B (2025a). "Mixed-Effects Additive Transformation Models with the R Package **tramME**." *Journal of Statistical Software*, **114**(11). doi:10.18637/jss.v114.i11.

Tamási B (2025b). ***tramME**: Transformation Models with Mixed Effects.* doi:10.32614/CRAN.package.tramME. R package version 1.0.8.

Tamási B, Hothorn T (2021). "**tramME**: Mixed-Effects Transformation Models Using Template Model Builder." *The R Journal*, **13**(1), 581–594. doi:10.32614/RJ-2021-075.

Therneau TM (2024a). ***coxme**: Mixed Effects Cox Models.* doi:10.32614/CRAN.package.coxme. R package version 2.2-22.

Therneau TM (2024b). ***survival**: Survival Analysis.* doi:10.32614/CRAN.package.survival. R package version 3.8-3.

Therneau TM, Grambsch PM, Pankratz VS (2003). "Penalized Survival Models and Frailty." *Journal of Computational and Graphical Statistics*, **12**(1), 156–175. URL http://www.jstor.org/stable/1391074.

Wood S (2025). ***mgcv**: Mixed GAM Computation Vehicle with Automatic Smoothness Estimation.* doi:10.32614/CRAN.package.mgcv. R package version 1.9-4.

Wood S, Pya N, Säfken B (2016). "Smoothing Parameter and Model Selection for General Smooth Models (with Discussion)." *Journal of the American Statistical Association*, **111**, 1548–1575. doi:10.1080/01621459.2016.1180986.

Zhou J, Zhang J, Lu W (2022a). "**TransModel**: An R Package for Linear Transformation Model with Censored Data." *Journal of Statistical Software*, **101**(9), 1–12. doi:10.18637/jss.v101.i09.

Zhou J, Zhang J, Lu W (2022b). ***TransModel**: Fit Linear Transformation Models for Right Censored Data*. doi:10.32614/CRAN.package.TransModel. R package version 2.3.

Affiliation:

Sandra Siegfried, Bálint Tamási, and Torsten Hothorn
Institut für Epidemiologie, Biostatistik und Prävention
Universität Zürich
Hirschengraben 84, CH-8001 Zürich, Switzerland
Siegfried.Sandra@protonmail.ch, Torsten.Hothorn@uzh.ch
